# Supplementary material for: Sustainability of Transient Kinetic Regimes and Origins of Death
Source: Sci Rep. 2016 Feb 8;6:20562. doi: 10.1038/srep20562 (PMC4744936; doi:10.1038/srep20562)
Supplement: Supplementary Information [file srep20562-s1.pdf]

Supporting Information for:
Sustainability of Transient Kinetic Regimes and Origins of Death

Dmitry Yu. Zubarev[1] and Leonardo A. Pachón[2]

[1] *Department of Chemistry and Chemical Biology, Harvard University, Cambridge, MA 02138 USA.*

[2] *Grupo de Física Atómica y Molecular, Instituto de Física, Facultad de Ciencias Exactas y Naturales, Universidad de Antioquia UdeA; Calle 70 No. 52-21, Medellín, Colombia.*

(Dated: October 10, 2015)

I. KINETIC SIMULATION OF COMPARTMENTALIZED COUPLED OSCILLATORS

$$k_1 = 0.007;$$
$$k_{-1} = 0.0001;$$
$$k_2 = 0.01;$$
$$k_{-2} = 0.0001;$$
$$k_3 = 1;$$
$$k_{-3} = 0.0001;$$
$$k_4 = 1;$$
$$k_{-4} = 0.0001;$$

Numerical integration carried out in MatLab R2014.a. using standard numerical integrator "ode45" with integration step 0.005 over 100000 steps.

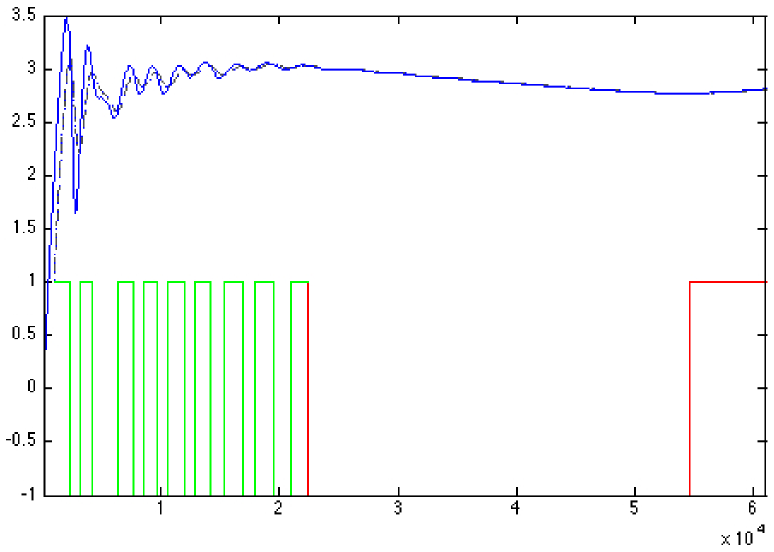

FIG. 1. Operational estimation of the lifetime of the oscillatory regime. The solid line is the concentration time-series for the intermediate Y obtained from the kinetic simulations; the dashed grey line is its moving-average.

We evaluate the sign function of the difference between the raw and the averaged time-series:

$$\begin{aligned} \text{sign}(a-b) &= 1, a > b, \\ \text{sign}(a-b) &= -1, a < b. \end{aligned} \tag{1}$$

Oscillation is considered to persist for as long as the sign function changes the value. We introduce a threshold on the ratio between two consecutive intervals for the change of sign in order to eliminate contamination due to slow non-monotonous concentration changes. In this example, the solid red line and solid green line are plots of the sign function. The former picks up contribution from a a slow non-monotonous concentration change and yields longer estimates of the oscillation lifetimes. Instead, we use the truncated data (green line) to obtain lifetime values.

II. EFFECTIVE ENVIRONMENT PERSPECTIVE

Derivation of the expression for noise in the limit of the nearest neighbor contribution:

$$\dot{A} = -k_1 A + k_{-1} X, \tag{2}$$

$$\dot{B} = -k_2 B + k_{-2} DY, \tag{3}$$

$$\dot{D} = k_2 BX - k_{-2} DY, \tag{4}$$

$$\dot{E} = k_4 X - k_{-4} E, \tag{5}$$

$$\dot{X} = k_1 A - k_{-1} X - k_{-2} DY + k_3 X^2 Y - k_{-3} X^3 - k_4 X + k_{-4} E, \tag{6}$$

$$\dot{Y} = k_2 BX - k_{-2} DY - k_3 X^2 Y + k_3 X^3 + \sum_n c_n (Y_n - Y), \tag{7}$$

$$\dot{A}_n = -k_1 A_n + k_{-1} X_n, \tag{8}$$

$$\dot{B}_n = -k_2 B_n + k_{-2} D_n Y_n, \tag{9}$$

$$\dot{D}_n = k_2 B_n X_n - k_{-2} D_n Y_n, \tag{10}$$

$$\dot{E}_n = k_4 X_n - k_{-4} E_n, \tag{11}$$

$$\dot{X}_n = k_1 A_n - k_{-1} X_n - k_{-2} D_n Y_n + k_3 X_n^2 Y_n - k_{-3} X_n^3 - k_4 X_n + k_{-4} E_n, \tag{12}$$

$$\dot{Y}_n = k_2 B_n X_n - k_{-2} D_n Y_n - k_3 X_n^2 Y_n + k_3 X_n^3 - c_n (Y - Y_n), \tag{13}$$

$$\begin{aligned} &\ddot{Y} - k_2 \dot{B} X - k_2 B \dot{X} + k_{-2} \dot{D} Y + k_{-2} D \dot{Y} + 2k_3 X \dot{X} Y + k_3 X^2 \dot{Y} + 3k_3 X^2 \dot{X} + \dot{Y} \sum_n c_n^2 - Y \sum_n c_n^2 \\ &= \sum_n c_n \left(k_2 B_n X_n - k_{-2} D_n Y_n - k_3 X_n^2 Y_n + k_3 X_n^3 - c_n Y_n \right), \end{aligned} \tag{14}$$

$$\begin{aligned} &\ddot{Y} - k_2 \dot{B} X - k_2 B \dot{X} + \left[k_{-2} \dot{D} + 2k_3 X \dot{X} - \sum_n c_n^2 \right] Y + \left[k_{-2} D + k_3 X^2 + \sum_n c_n^2 \right] \dot{Y} + 3k_3 X^2 \dot{X} \\ &= \sum_n c_n \left(k_2 B_n X_n - k_{-2} D_n Y_n - k_3 X_n^2 Y_n + k_3 X_n^3 - c_n Y_n \right), \end{aligned} \tag{15}$$

Because the term at the right-hand side does not depend functionally on Y, it can be assumed to be a given function of time $\xi(t)$.

$$\begin{aligned} \ddot{Y} - k_2 \dot{B} X - k_2 B \dot{X} + &\left[k_{-2} \dot{D} + 2k_3 X \dot{X} - \sum_n c_n^2 \right] Y \\ &+ \left[k_{-2} D + k_3 X^2 + \sum_n c_n^2 \right] \dot{Y} + 3k_3 X^2 \dot{X} = \xi(t) \end{aligned} \tag{16}$$

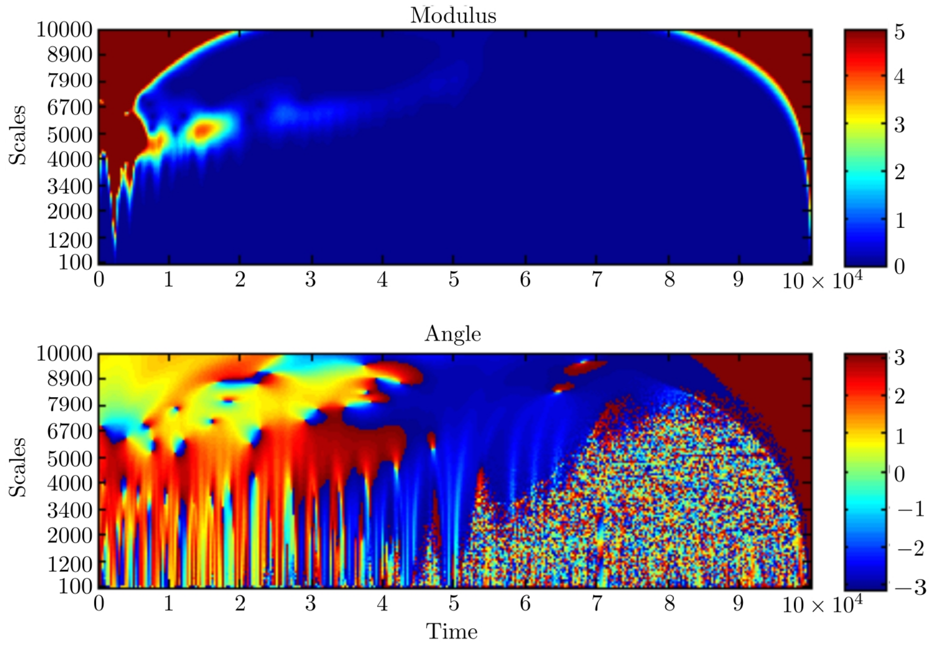

FIG. 2. Wavelet cross spectrum for the time series of c_Y oscillations in the compartment and its effective environment noise (Fig. 5 in the main text). The top panel shows the magnitude of the wavelet cross spectrum; the bottom panel shows the wavelet coherence. Both signals have significant contribution between scales 5600 and 6700 over the interval [0;50000].
